# Aerobic exercise 3 times per week in adult rats did not influence the progression of periodontal disease

**DOI:** 10.3389/fspor.2023.1238500

**Published:** 2023-10-23

**Authors:** Thalita Borges Souza, Rafael Reimann Baptista

**Affiliations:** ^1^ Postgraduate Program in Biomedical Gerontology, School of Medicine, PUCRS, Porto Alegre, Brazil; ^2^ Postgraduate Program in Dentistry, School of Health and Life Sciences, PUCRS, Porto Alegre, Brazil

**Keywords:** periodontitis, swimming, physical exercise, wistar, inflammation

## Abstract

**Introduction:**

Physical exercise has proven efficacy in the prevention and treatment of chronic diseases, and its anti-inflammatory effect has been evaluated as a potential preventive factor in the progression of periodontal disease, in addition to improving physiological parameters.

**Methods:**

To test this hypothesis regarding its preventive factor, we evaluated the effects of aerobic training on the progression of periodontal disease in 8-month-old Wistar rats (*n* = 44). The animals underwent a swimming protocol lasting six weeks, with periodontal disease induced by ligature in the fifth week, totaling fourteen days of ligature placement. Anthropometric parameters were measured for subsequent calculations of BMI and Lee's Index. Interleukin-1β testing was performed to measure serum inflammatory parameters, and alveolar bone loss was measured using images to calculate the area of loss.

**Results:**

The trained animals showed no significant differences compared to the non-trained animals in terms of anthropometric measures. Regarding the area of bone loss, although there were significant differences between the groups with and without periodontal disease, exercise did not demonstrate an impact on rats with the disease. IL-1β analysis did not detect any measurable values in the samples in either group.

**Discussion:**

These findings indicate that the applied exercise protocol was not sufficient to attenuate the progression of periodontal disease. This study did not find an effective impact of physical exercise on the analyzed parameters; however, the results are important in highlighting that the experimental animal model for inducing periodontal disease is efficient, which may encourage further investigations to determine factors that can attenuate its progression. Similarly, the application or development of new exercise protocols that can benefit and enrich the discussion on its positive effects in this disease is important, as there is already evidence suggesting an effective relationship between exercise and disease progression.

## 1. Introduction

Periodontal disease (PD) is an inflammatory disease that affects the supporting structures of the teeth as a result of the interaction between a pathogenic microbiota and the host's immune response (1), leading to loss of attachment as it progresses. The main conditions of periodontal disease include gingivitis and periodontitis, which are characterized by inflammatory and immune reactions to bacterial plaque. At times, these host defense reactions can be harmful, as they can damage neighboring cells and structures of the connective tissue in the presence of antibodies against periodontal pathogens (2).

Due to its wide occurrence, PD represents a significant problem for public health. This oral condition, which can result in tooth loss, impacts mastication, functional abilities, and aesthetics, causing a detriment to quality of life (3). Studies demonstrate a positive correlation between body mass index and periodontal disease (4–6). Obesity is a chronic metabolic disease that predisposes individuals to several comorbidities and is currently an important public health problem. It is associated with systemic inflammation and may be a risk factor for periodontal disease (7, 8). Additionally, it is significantly associated with non-communicable chronic diseases of aging, such as type 2 diabetes (9). There is evidence showing a considerable increase in the prevalence of PD with advancing age (10).

Thus, the inflammation resulting from periodontal disease can play an important role in systemic health, as various inflammatory markers are released into the bloodstream, potentially being a risk factor for systemic and cardiovascular diseases (11). To understand this process, interdisciplinary knowledge aiming at the comprehensive health of the individual is important (12).

Also, inflammation and oxidative stress are related to the genesis process of the periodontal disease, although the mechanisms underlying this relationship are not fully understood. In this sense aerobic training is an important non-pharmacological treatment that attenuates inflammation (13) and oxidative stress (14) even though with a moderate effect.

The beneficial effect of physical exercise on chronic and inflammatory diseases is constantly reported in the literature, either as a protective effect and/or as an attenuating factor in disease progression. For example, after 12 weeks of combined aerobic and strength training, obese men showed positive outcomes in periodontal improvement, as well as a reduction in obesity-related complications (15). Despite the evidence regarding the antioxidant and anti-inflammatory effects of aerobic training (13) in previous studies, little has been explored regarding the mechanisms of this relationship and variables such as physical activity level and age in periodontal disease. Therefore, it becomes relevant for this research field to test new training protocols aimed at establishing the necessary level to contribute to the attenuation of the inflammatory process of periodontal disease. Swimming is widely used in experiments with rats, as it is a natural skill that greatly reduces stress and anxiety, minimizing their influence on the effects of applied treatments (16). One of the previously researched positive effects of aerobic training on PD is the attenuation of alveolar bone loss (17), reducing the inflammatory process and, thus, attenuating its progression. Therefore, the aim of this study was to evaluate the impact of aerobic training on the progression of periodontal disease in adult rats, specially trying to advance our knowledge regarding the effects of exercise in PD and its relationship between alveolar bone mass evaluation, IL-1β, BMI and the underlying aspects related to the volume intensity relationship of the aerobic training.

## 2. Material and methods

### 2.1. Animals

Forty-four male Wistar rats (Rattus norvegicus albinus), eight months old, were used in this study. The rats were provided by the Experimental Biological Models Center of PUCRS (CEMBE, PUCRS; Porto Alegre, RS). The animal procedures were carried out between 8 am and 6 pm, with the laboratory temperature controlled at 22–24°C. Initially, the rats were subjected to a two-week acclimation period to the room where the experiment would be conducted and to the individuals involved. This study was approved by the Ethics Committee for Animal Use (CEUA) of the Pontifical Catholic University of Rio Grande do Sul under number 10,598.

The animals were randomly divided into four groups, with 11 animals each (Table 1), considering the minimum number for ethical reasons while still providing sufficient statistical significance. The sample size was determined to provide 85% power to detect a significant difference of 15% between the groups, assuming a standard deviation of 15%, with a 95% confidence interval (α = 0.05%).

**Table 1:** Experimental groups.

Experimental groups	*n*
Group 1—sedentary without periodontal disease	11
Group 2—sedentary with periodontal disease	11
Group 3—exercise without periodontal disease	11
Group 4—exercise with periodontal disease	11

### 2.2. Anthropometric data

For the monitoring of anthropometric data, the weight (g) was measured weekly, and the nose-anal length (cm) of the animals was measured at the beginning and end of the experiment, respectively, using a digital scale with gram precision and a measuring tape. To measure the length, the animals were anesthetized with a small dose of intraperitoneal anesthetic (ketamine 30 mg/kg and xylazine 4 mg/kg). Additionally, for the calculation of obesity, the body mass index (BMI) and Lee's Index were used, following the protocol adopted by Quirós-Cognuck et al. (18). The BMI is calculated by dividing the weight in grams by the square of the length in cm, and Lee's Index is calculated as the cubic root of the body weight in grams divided by the length in cm.

### 2.3. Physical training

After the acclimation period, the animals in the groups underwent adaptation to the aquatic environment for two weeks: during the first week, they remained in shallow water (5 to 7 cm in depth) for 15 min per day, for five days. In the second week, the water depth was increased to 60 cm, starting with 10 min and gradually increasing by 10 min per day until reaching 60 min of uninterrupted exercise without load (19, 20). The animals that did not undergo training remained in the shallow water during this adaptation period.

**Figure 1:**
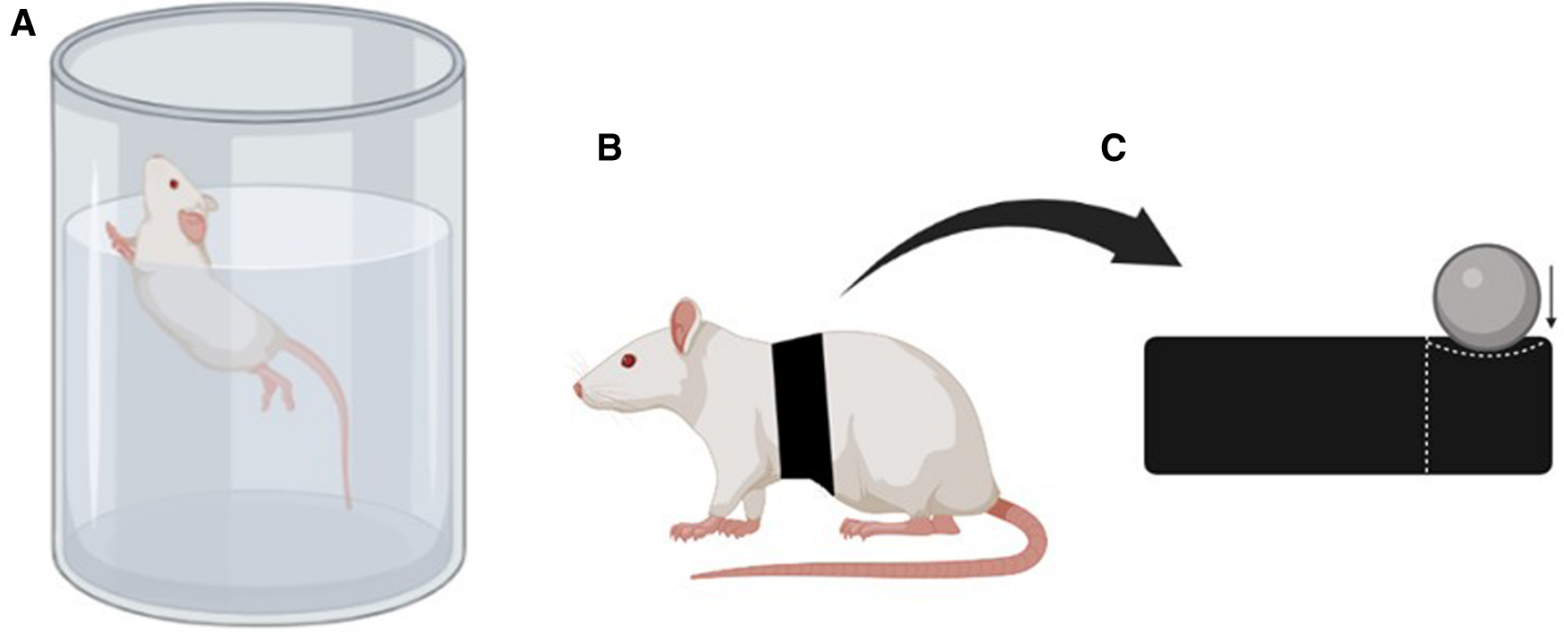
(**A**) Representation of the performed physical exercise, (**B**) representation of the animal with the elastic band fixed to the back, and (**C**) representation of the elastic band with a compartment for adding weight (created with BioRender.com).

The aerobic training program consisted of six weeks of swimming training as proposed by Rosety-Rodriguez et al. (21). The rats swam individually in plastic tanks (130 cm in diameter and 80 cm in height) (Figure 1A) for 60 min per day, on 3 alternate days per week. The water depth was maintained at 60 cm to prevent the animals from resting their tails on the bottom of the tank. During the swimming sessions, the rats wore elastic thoracic bands to which adjustable weights could be added (Figures 1B,C). The rats started exercising without any additional load in the first week, and from the second week of training, 3% of their body weight was added, increasing by an additional 1% each week until the end of the study. The lead weights were adjusted according to the weekly body weight. If an animal showed signs of exhaustion, such as being unable to swim to the water surface for 10 s, they were removed from the water for a five-minute rest period. To minimize the stress associated with water exposure, the water temperature was maintained between 30 and 32°C. After each animal completed the training, the water temperature dropped by approximately 0.5 to 1°C, and it was stabilized as needed before the next animal began swimming.

To separate the effects of exercise from environmental stress, the rats in the non-exercised groups were individually placed in identical swimming tanks but remained in shallow water without sufficient depth for swimming movements, at the same temperature and frequency as the exercised rats, for 30 min. Occasionally, some rats exhibited floating behavior, requiring gentle water agitation or a light touch with fingers on the animal's back to ensure active swimming. The animals' bodies and tails were dried with an absorbent towel immediately after exiting the swimming tank, and they were then returned to their cages.

### 2.4. Induction of periodontal disease

**Figure 2:**
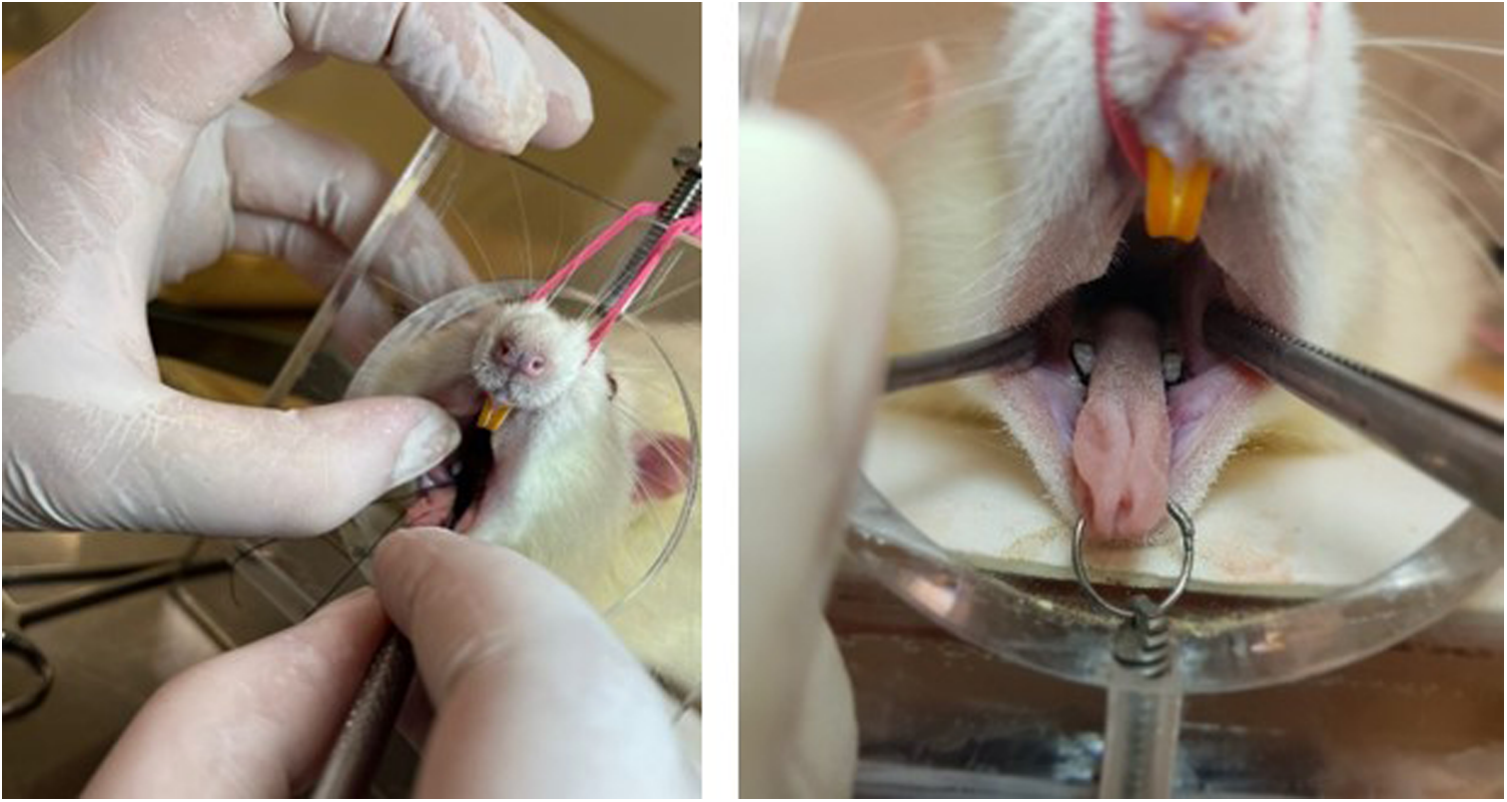
Induction of periodontal disease by ligature.

The induction of periodontal disease was performed fourteen days before the end of the experiment using the ligature method (22) with 4–0 silk thread (Ethicon), sterilized, around the first lower molar in the left and right jaws (Figure 2). The ligatures were placed in a subgingival position. The procedure was performed under general anesthesia using a mixture of ketamine (80 mg/kg) and xylazine (10 mg/kg) intraperitoneally (23). During this period, a sterile gauze soaked in saline solution was placed over the eyes to prevent drying. The laboratory temperature was maintained between 22 and 24°C to prevent hypothermia. The animals were returned to the vivarium only after complete anesthetic recovery. In all animals, the ligature was inspected one week after placement and repositioned if necessary, totaling 14 days with the ligature. The rats in the trained groups were not subjected to exercise 24 h before and after ligature placement.

### 2.5. Euthanasia

The animals were euthanized 24 h after the last exercise session, under anesthesia, with intraperitoneal injection of ketamine (80 mg/kg) and xylazine (10 mg/kg), and subsequently sacrificed by exsanguination through cardiac puncture. Blood samples were centrifuged to obtain serum and stored frozen at −80°C until analysis. Additionally, the mandibles were removed and stored in 10% formaldehyde.

### 2.6. Alveolar bone mass evaluation

After being immersed in formaldehyde for 48 h, the mandibles were washed in distilled water and soaked in 3% hydrogen peroxide solution to remove soft tissues. Any remaining tissues were mechanically removed. The mandible was then divided in half along the midline and between the central incisors. The specimens were stained with 1% aqueous methylene blue (22) to differentiate bone from teeth. The lingual aspect of the first molars was photographed using a 16× magnification. The images were measured using computer software (Image J), which calculated the area of bone loss. The average of three measurements was used as the final value.

### 2.7. Measurement of IL-1β

IL-1β in serum was measured using an enzyme-linked immunosorbent assay (ELISA) kit from R&D Systems. The detection range of the assay was between 62.5 and 4,000 pg/ml. The test was performed according to the manufacturer's specifications. Analysis of the samples was conducted by measuring absorbance at a wavelength of 450 nm with correction at 540 nm using an ELISA reader. Due to the limited availability of assay kits, a total of 36 rats were tested, with nine rats in each group. In groups 1 and 3, two rats were randomly selected for analysis. In group 2, one rat was excluded due to a broken molar that rendered the analysis impossible, and another rat was randomly selected instead. In group 4, two rats died, leaving nine rats available for analysis.

### 2.8. Statistics

The normality of the data was assessed using the Shapiro-Wilk test (*P* > 0.05), and the results were expressed as mean ± standard deviation. GraphPad Prism software (version 8.4.2) was used to analyze the raw data. For the analysis of BMI, Lee Index, and alveolar bone loss data, a one-way ANOVA test followed by Tukey's *post hoc* test was applied for multiple comparisons between groups to determine any significant differences, using a significance level of 5%. For IL-1β data, a simple linear regression test was used with a significance level of <0.0001, and sample interpolation was performed using the standard curve.

## 3. Results

### 3.1. Body mass index (BMI) and lee index

There were no significant differences among the four study groups comparing the beginning to the end of the experiment. Two animals died during the experiment, and therefore, the data from these two animals were excluded. The cutoff point for the BMI indicator was proposed to be between 0.45 and 0.68 g/cm² (24). In this study, all rats started and finished the experiment above this value, with mean values of 0.83 ± 0.08 pre-training and 0.80 ± 0.07 post-training. Meanwhile, the Lee Index has a cutoff value of 0.30 to determine obesity (25), which was essentially the average obtained in these results (Table 2).

**Table 2:** Dados antropométricos.

	Group 1		Group 2		Group 3		Group 4	
Parameters	Pre	Post	Pre	Post	Pre	Post	Pre	Post
Body weight (g)	660.70 ± 81.99	674.4 ± 88.32	647.2 ± 82.78	638.0 ± 78.51	634.5 ± 82.11	642.0 ± 78.12	635.9 ± 71.63	617.90 ± 73.49
Body length (cm)	27.55 ± 1,21	28.64 ± 0.92	27.91 ± 1.04	28.18 ± 1.25	28.09 ± 1.14	28.19 ± 1.04	27.73 ± 0.94	28.56 ± 0.88
BMI (g/cm²)	0.87 ± 0.07	0.83 ± 0.07	0.83 ± 0.08	0.80 ± 0.06	0.80 ± 0.06	0.81 ± 0.06	0.82 ± 0.82	0.75 ± 0.06
Lee index	0.32 ± 0.01	0.31 ± 0.01	0.31 ± 0.01	0.30 ± 0.01	0.31 ± 0.01	0.31 ± 0.01	0.31 ± 0.01	0.30 ± 0.01

### 3.2. Alveolar bone loss

**Figure 3:**
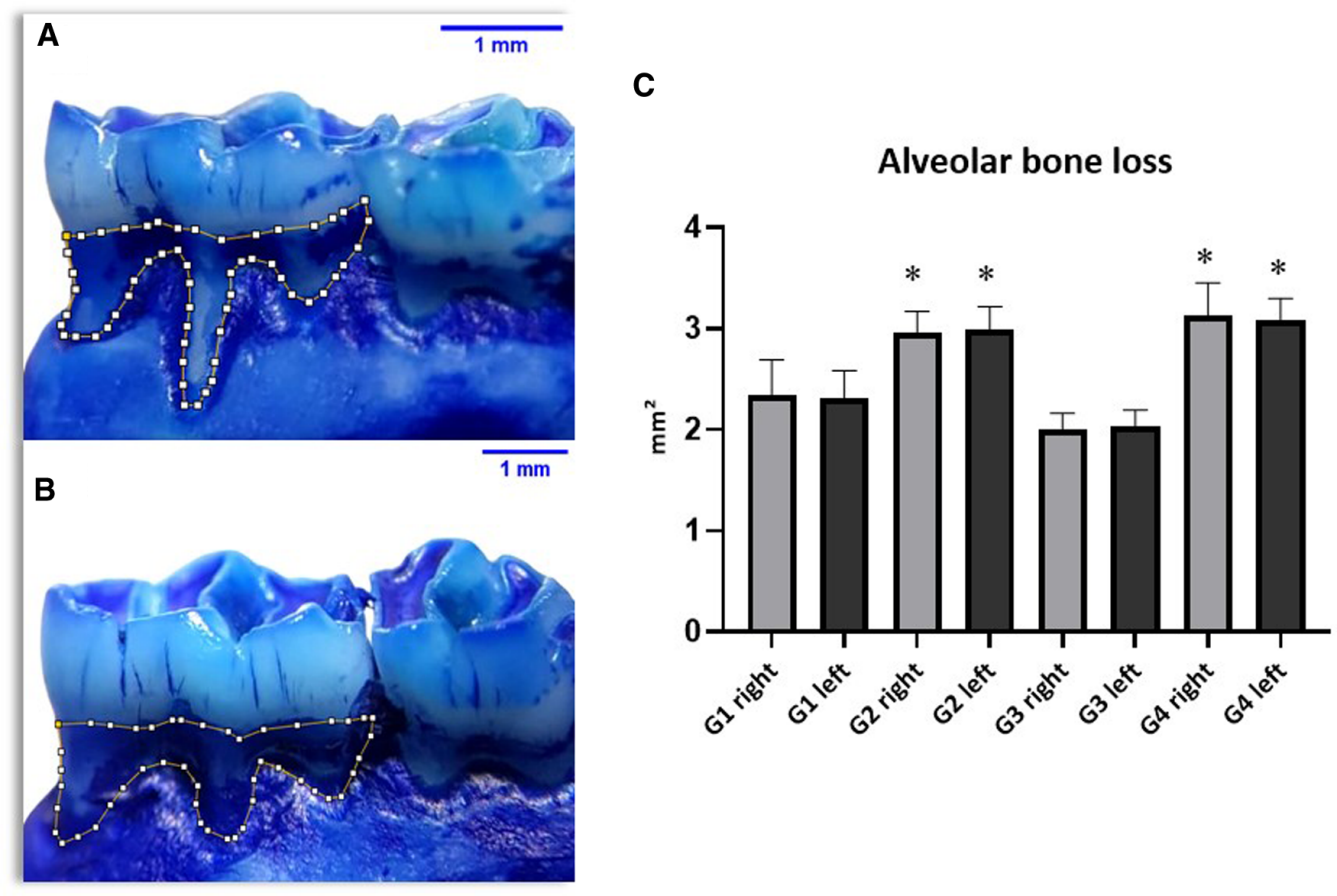
Results of alveolar bone loss (**AB**) showing significant differences between groups (**C**) with periodontal disease (G2 and G4) and groups without the disease (G1 and G3).

It can be observed that the evaluated alveolar bone loss showed significant differences in the groups with induced periodontal disease, thus demonstrating the effectiveness of the experimental model. However, when comparing these groups (G2 and G4) trained with periodontal disease to the non-trained groups, no significant difference was found, as shown by the mean results of the periodontal disease groups: G2 presenting 2.97 ± 0.36 mm^2^ on the right side and 3.06 ± 0.58 mm^2^ on the left side; G4 presenting 3.08 ± 0.50 mm^2^ on the right side and 3.26 ± 0.39 mm^2^ on the left side (Figure 3).

### 3.3. IL-1β

**Figure 4:**
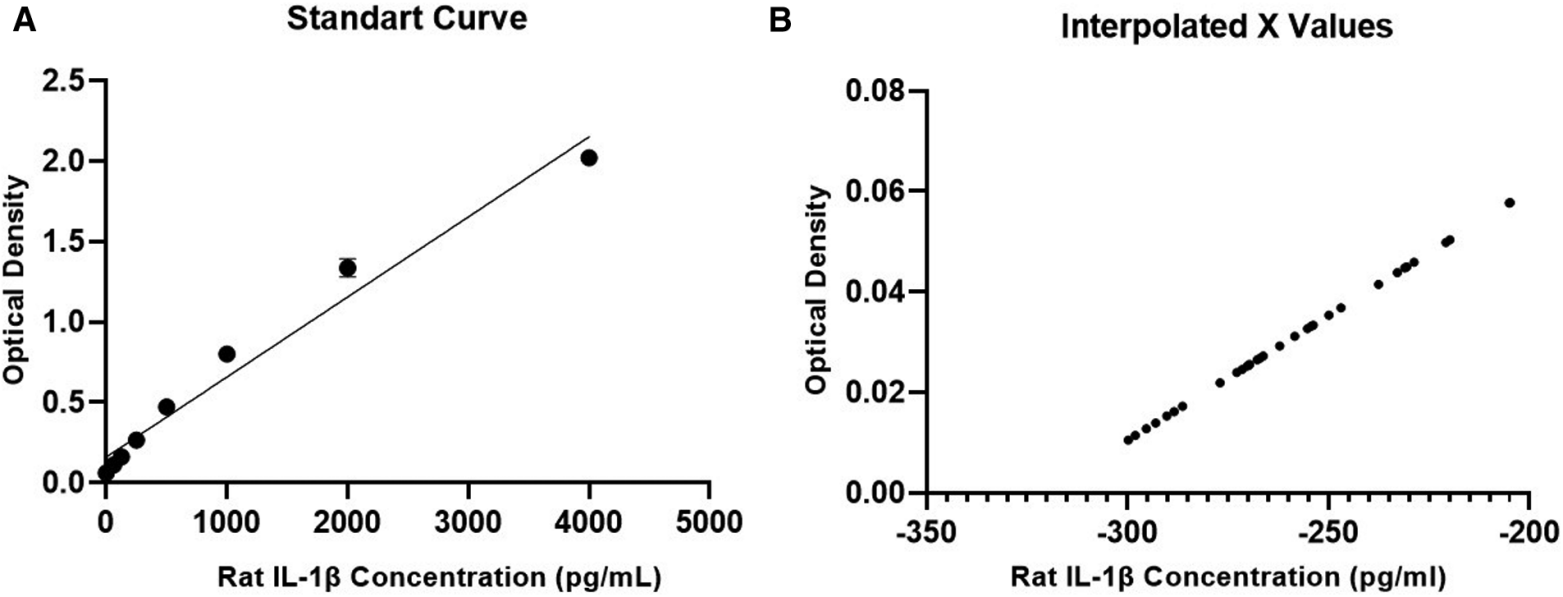
IL-1β concentration results. (**A**) Standard calibration curve for the test, (**B**) curve with results of the tested samples.

Regarding IL-1β, the standard curve was generated for comparison with the set of tested samples. Both curves generated from the test showed an R-squared value > 0.95, demonstrating the quality of the assay performed. However, when measuring IL-1β levels in serum samples, no detectable values were obtained, and all results were negative (Figure 4). In other words, none of the groups showed detectable levels of the interleukin, indicating that a systemic inflammatory process was not evident in the samples using this method.

## 4. Discussion

The main finding of this study was that the investigated aerobic training protocol did not attenuate the progression of periodontal disease, as evidenced by the clinical evaluation of alveolar bone loss and the analysis of the inflammatory profile using the IL-1β test. Additionally, the anthropometric parameters evaluated through BMI and Lee Index also did not show any significant differences among the groups (Table 2).

This result was surprising, considering that several studies in the literature have demonstrated the beneficial effects of physical training on the inflammatory process (12, 23, 26). Exercise normally triggers metabolic shifts that can affect inflammation, such as changes in nutrient signaling pathways like mTOR and SIRT1. However, the rats' obesity could have resulted in metabolic inflexibility, making these pathways less responsive to the potential anti-inflammatory effects of exercise (27).

Another aspect is that gut microbiome can influence systemic inflammation. The near obesity in our rats could have led to a dysbiotic microbiome, which could negate the anti-inflammatory effects of exercise. Exercise has been shown to modulate the gut microbiota, but if the dysbiosis is severe, exercise alone may not suffice (28).

The induction of periodontal disease using ligature is widely reported in the literature (20, 29, 30) due to its resemblance to chronic periodontitis in humans, as alveolar bone resorption depends on oral bacterial load and the gingival tissue becomes infiltrated with inflammatory cells. This model is considered suitable for analyzing gingival tissue inflammation with changes related to systemic diseases. Animal models of periodontal disease are very useful in elucidating the molecular pathways involved in new treatment strategies. Additionally, in human studies, it is difficult to control environmental and social variables to establish a cause-and-effect measure of treatment outcomes. Furthermore, according to previous studies, ligature placement for 14 days seems to be sufficient to result in alveolar bone loss (20).

In this study, the alveolar bone loss was observed through clinical analysis, but exercise did not have a protective effect on its progression. The lack of attenuation in disease progression may be related to the duration of exercise or insufficient training sessions to generate a protective factor. Previous studies have described protocols involving training for five or six days per week and a training period exceeding six weeks (19, 31, 32). However, a very popular weekly frequency among exercise practitioners is three times per week, which motivated us to study the effects of such a frequency.

Another relevant aspect to justify the lack of beneficial results from exercise in our study may have been the fact that the rats were sedentary until the eighth month of life and were also above the cutoff point indicating obesity, which may have interfered with their swimming performance.

Given that the rats were sedentary until the eighth month of life, cellular senescence might be a factor to explain the absence of effects from the exercise. Senescent cells are less responsive to anti-inflammatory stimuli, including those potentially triggered by exercise (33). Furthermore, exercise is known to release anti-inflammatory hormones like cortisol, which can reduce inflammation. However, the physiological stress of obesity and sedentary lifestyle could have affected the hormonal balance in such a way that it counteracted the anti-inflammatory benefits usually associated with exercise (34).

After the addition of load to the training, some rats were unable to maintain the same performance during the 60-min exercise session, requiring adaptation of the training by adding breaks or even terminating the session if the animal could no longer continue.

Obesity has been the focus of another study, where obese rats showed higher densities of polymorphonuclear leukocytes in the gums compared to normal rats, indicating that obesity can be a risk factor for periodontal diseases (4). This characteristic of our study can be considered interesting in terms of clinical applications, as a significant portion of the population is sedentary and experiencing obesity.

Another relevant point is that the aim of the study was to perform low to moderate intensity aerobic training, which influenced the decision not to allow the rats to reach exhaustion during exercise. Perhaps the protocol used was not the most suitable for these animals. One option would be to increase the training volume and remove the added load to the body weight, that is, reducing the intensity, as has been done in other studies (31, 32, 35). The choice of this protocol was based in the literature that shows that results can already be observed after six weeks of training (21, 32), and also considering the application of this protocol in older rats who are not capable of performing high-intensity exercises. Since periodontal disease more effectively affects this population, it becomes interesting to study both the effects of the disease and exercise in older rats.

The swimming is considered a less traumatic exercise for animals because, in addition to animals having an innate ability to swim, it has the advantage of not causing foot injuries as in the case of treadmill running and climbing according to the Resource book for the design of animal exercise protocols (36). Furthermore, swimming provides a more uniform type of physical activity when conducted under ideal conditions, such as exercise intensity and volume, water temperature, and water depth. Aerobic training, which is characterized by high volume and low/moderate intensity, is commonly used in swimming studies with rats, both with and without the addition of load relative to the animals' body mass. However, considering what was previously mentioned regarding the heterogeneity of rat populations and individual heterogeneity, the same exercise can have different intensities among individuals.

During the periodontal disease progression, circulating levels of pro-inflammatory cytokines increase, including interleukins (IL-1β, IL-2, IL-6, IL-8) (25). IL-1β, along with TNF-α, has the potential to trigger inflammatory reactions that lead to periodontal bone resorption and destruction (37). These concentrations can be measured in the blood or gingival tissue, allowing for a comparison between healthy mucosa and mucosa affected by periodontal disease through their quantification (23).

The limitations of this study regarding the analysis of this inflammatory marker (IL-1β) are related to the inability to compare it with other studies where the analysis was conducted in buccal tissue rather than blood, using the same detection range (38). Systemic detection may demonstrate lower accuracy for detection, as well as a lower presence of significant differences in the samples (29). Conversely, determining local cytokine levels in terms of measuring the local inflammatory condition may provide more reliable results (39). Additionally, by inducing the condition in only two teeth, we may have caused a reduced systemic impact.

Furthermore, the analysis of only one pro-inflammatory marker limited the search for a consistent pattern. The cause-effect relationship between cytokines and the loss of periodontal tissue has been reported in the literature, so the addition of more cytokines in the analysis, both pro-inflammatory and anti-inflammatory, could provide a broader understanding of the systemic inflammatory process.

Additionally, considering a more comprehensive framework for the physiological evaluation of the impact of physical training in rats, tests involving muscle assessment, such as improvements in the antioxidant system in skeletal muscle (21), can provide important data for this evaluation. Aerobic training increases the production of reactive oxygen species (ROS), which in turn activates antioxidant defense mechanisms. However, it is important to consider that conditions like inflammatory disease, sedentarism, obesity and aging, are conditions that might already involve high levels of ROS, negating any additional anti-oxidative benefits from exercise (40) and thus explain the mild effects of training in our study.

Furthermore, metabolic tests with blood markers (41) can demonstrate other systemic adaptations to exercise and disease, and help determine the type of physical training (aerobic and anaerobic) that best addresses the needs of analyzing its effects.

In conclusion, the present study did not find an effective impact of physical training on the analyzed parameters. However, it reinforces the effectiveness of the animal experiment with rats inducing periodontal disease, and the need to test other exercise protocols for this specific population in order to use them for modulating the inflammatory response, as well as for protection against chronic conditions such as obesity. Considering this specific population, from adulthood to senility, a protocol without added weights but with a higher training volume seems to be more suitable. Additionally, an increase in the number of weeks may be more beneficial in the overall condition of the animals and yield more results. Testing other inflammatory markers such as TNF-α, IL-6, and IL-10, which are widely observed in the literature (23, 42), can provide a more comprehensive understanding of the disease process.

## Data Availability

The raw data supporting the conclusions of this article will be made available by the authors, without undue reservation.
